# Protein synthesis rates of muscle, tendon, ligament, cartilage, and bone tissue *in vivo* in humans

**DOI:** 10.1371/journal.pone.0224745

**Published:** 2019-11-07

**Authors:** Joey S. J. Smeets, Astrid M. H. Horstman, Georges F. Vles, Pieter J. Emans, Joy P. B. Goessens, Annemie P. Gijsen, Janneau M. X. van Kranenburg, Luc J. C. van Loon

**Affiliations:** 1 Department of Human Biology, NUTRIM School of Nutrition and Translational Research in Metabolism, Maastricht University Medical Centre+, Maastricht, The Netherlands; 2 Department of Orthopedic Surgery, Maastricht University Medical Centre+, Maastricht, The Netherlands; University of Houston, UNITED STATES

## Abstract

Skeletal muscle plasticity is reflected by a dynamic balance between protein synthesis and breakdown, with basal muscle tissue protein synthesis rates ranging between 0.02 and 0.09%/h. Though it is evident that other musculoskeletal tissues should also express some level of plasticity, data on protein synthesis rates of most of these tissues *in vivo* in humans is limited. Six otherwise healthy patients (62±3 y), scheduled to undergo unilateral total knee arthroplasty, were subjected to primed continuous intravenous infusions with L-[ring-^13^C_6_]-Phenylalanine throughout the surgical procedure. Tissue samples obtained during surgery included muscle, tendon, cruciate ligaments, cartilage, bone, menisci, fat, and synovium. Tissue-specific fractional protein synthesis rates (%/h) were assessed by measuring the incorporation of L-[ring-^13^C_6_]-Phenylalanine in tissue protein and were compared with muscle tissue protein synthesis rates using a paired *t* test. Tendon, bone, cartilage, Hoffa’s fat pad, anterior and posterior cruciate ligament, and menisci tissue protein synthesis rates averaged 0.06±0.01, 0.03±0.01, 0.04±0.01, 0.11±0.03, 0.07±0.02, 0.04±0.01, and 0.04±0.01%/h, respectively, and did not significantly differ from skeletal muscle protein synthesis rates (0.04±0.01%/h; *P*>0.05). Synovium derived protein (0.13±0.03%/h) and intercondylar notch bone tissue protein synthesis rates (0.03±0.01%/h) were respectively higher and lower compared to skeletal muscle protein synthesis rates (*P*<0.05 and *P*<0.01, respectively). Basal protein synthesis rates in various musculoskeletal tissues are within the same range of skeletal muscle protein synthesis rates, with fractional muscle, tendon, bone, cartilage, ligament, menisci, fat, and synovium protein synthesis rates ranging between 0.02 and 0.13% per hour *in vivo* in humans.

**Clinical trial registration**: NTR5147

## Introduction

Skeletal muscle tissue plasticity is achieved by a dynamic equilibrium between muscle protein synthesis and breakdown rates. Temporary changes in either protein synthesis and/or protein breakdown result in net muscle protein accretion or loss. A routinely applied method to study skeletal muscle protein metabolism *in vivo* in humans is the continuous intravenous infusion of stable isotope labelled amino acids with frequent sampling of blood and skeletal muscle tissue using the percutaneous needle biopsy technique [1, 2]. This contemporary stable isotope methodology has been applied for several decades to show that skeletal muscle tissue turns over at a rate of approximately 1–2% per day [3]. Though widely applied in skeletal muscle research, and to a lesser extent in tendon research [4–13], there are very few data on *in vivo* protein synthesis rates of other musculoskeletal tissues in humans.

It is evident that other musculoskeletal tissues such as tendon, ligaments, bone, and cartilage should also possess a certain degree of plasticity. Damage due to injury or surgery generally involves much more tissues than merely skeletal muscle. Obviously, recovery and rehabilitation requires plasticity of all tissues involved. Several studies have assessed tendon protein synthesis rates *in vivo* in humans using stable isotope methodology [4–12, 14, 15]. Furthermore, emerging research is now establishing the relevance of intramuscular as well as extramuscular collagen structures being required for the proper transduction of force generated by muscle contraction [16, 17]. Clearly, connective tissue plasticity plays an important role in determining musculoskeletal strength and functional capacity [18, 19].

The application of contemporary stable isotope methodology to assess tissue protein synthesis rates in other musculoskeletal tissues such as tendon, ligaments, bone, and cartilage is restricted due to the obvious logistical and medical ethical restraints of tissue sampling. To omit these restrictions we selected 6 otherwise healthy male (*n* = 3) and female (*n* = 3) adults, scheduled to undergo unilateral total knee arthroplasty, to participate in a study in which we applied contemporary stable isotope methodology to assess basal protein synthesis rates of a wide variety of musculoskeletal tissues including muscle, tendon, ligament, bone, cartilage, menisci, fat, and synovium. We hypothesized that basal protein synthesis rates of various musculoskeletal tissues are different compared to skeletal muscle tissue.

## Materials and methods

### Subjects

Six otherwise healthy male (*n* = 3) and female (*n* = 3) adults (age: 62±3 y; body weight: 89.8±4.7 kg; body mass index: 28.6±1.1 kg/m^2^), scheduled to undergo unilateral total knee arthroplasty, were recruited to participate in the present study. Subjects had no history of participating in any stable isotope infusion studies prior to this experiment. Exclusion criteria included secondary osteoarthritis of the knee, the use of intra-articular corticosteroid injections or bisphosphonates within 3 months prior to surgery, previous surgical intervention of the knee, rheumatoid arthritis or other systemic inflammatory diseases, and collagen disorders (e.g. Marfan and Ehlers-Danlos). All subjects were informed about the nature and possible risks of the experimental procedures, before their written informed consent was obtained. The study was approved by the Medical Ethical Committee of Zuyderland Medical Centre, Heerlen, The Netherlands, and conformed to the principles outlined in the declaration of Helsinki for use of human subjects and tissue.

### Study design

**Fig 1:**
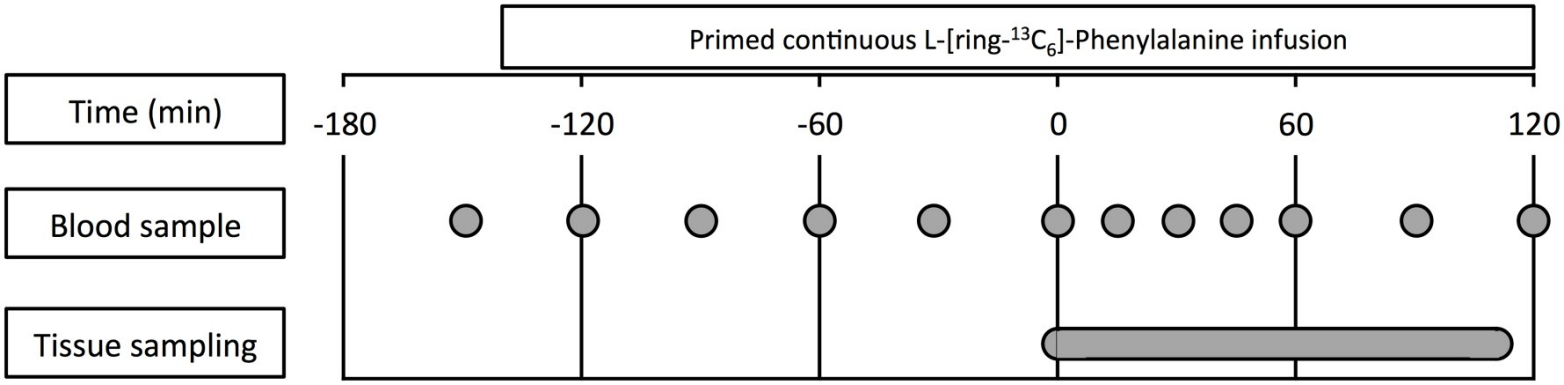
Schematic representation of the infusion protocol. *t* = 0 min represents the start of the surgical procedure.

The experimental protocol is outlined in Fig 1. Each subject was diagnosed with knee osteoarthritis and, therefore, underwent unilateral total knee arthroplasty at the Department of Orthopedic Surgery at Maastricht University Medical Centre+, The Netherlands. Before and during surgery patients were subjected to primed continuous intravenous infusions with L-[ring-^13^C_6_]-Phenylalanine. Four patients underwent general anesthesia and two patients underwent spinal anesthesia. Blood and tissue samples were collected throughout the surgical procedure to assess fractional muscle, tendon, ligament, bone, cartilage, menisci, fat, and synovium protein synthesis rates (FSR; %/h).

### Infusion protocol

All patients were fasted for at least 6 h prior to surgery. About 2.5 h before surgery a Teflon catheter was inserted into an antecubital vein for stable isotope infusion. A second Teflon catheter was inserted into a heated dorsal hand vein of the contralateral arm for perioperative blood sampling. After taking a baseline blood sample at *t* = -150 min, the serum phenylalanine pools were primed with a single dose of L-[ring-^13^C_6_]-Phenylalanine (2 μmol/kg), after which continuous intravenous L-[ring-^13^C_6_]-Phenylalanine (0.05 μmol/kg/min) infusion was initiated. Subsequently, blood samples were collected at *t* = -120, -90, -60, -30, 0 (start of the surgical procedure), 15, 30, 45, 60, 90, and 120 min (Fig 1).

To determine basal tissue protein synthesis rates, tissue samples of the *vastus lateralis* muscle, patellar tendon, femur, tibia, cruciate ligaments, femoral cartilage, menisci, synovium, and Hoffa’s fat pad were obtained throughout the surgical procedure. All tissue samples were collected through surgical excision, except for the *vastus lateralis* muscle which was collected from the middle region of the *vastus lateralis*, approximately 15 cm above the patella and 3 cm below entry through the fascia, using the standard percutaneous needle biopsy technique [20]. Conventional muscle biopsy samples of the *vastus lateralis* were collected to assess skeletal muscle protein synthesis rates as a reference to the synthesis rates of the various musculoskeletal tissues obtained during surgery. All tissue samples were obtained directly after opening the joint and no tourniquet was used. For tissues that were visually affected by the disease process, such as cartilage and bone, we ensured that only parts of the tissues were sampled which appeared unaffected and healthy. Since vascularization and nutrient supply may differ substantially between and within tissues [21–26], all tissues were sampled in such a way that the obtained material was representative for the tissue. For bone tissues a mixture of cortical and trabecular bone was sampled (except for the predominantly trabecular notch bone tissue), and for the fibrous and intra-articular soft tissues complete cross sectional samples were obtained. Hereafter, samples were freed from any visible blood, immediately frozen in liquid nitrogen, and stored at -80°C until subsequent analysis. In addition, blood samples were collected at frequent intervals to determine L-[ring-^13^C_6_]-Phenylalanine enrichment in serum protein. Blood samples were collected in serum tubes and centrifuged at 3500*g* for 15 min at 20°C to obtain serum. Aliquots of serum were frozen in liquid nitrogen and stored at -80°C. For a schematic representation of the infusion protocol, please see Fig 1.

### Serum analyses

Serum amino acid concentrations and enrichments were determined by gas chromatography-mass spectrometry (GC-MS; Agilent 7890A GC/5975C; MSD, Little Falls, DE), as described in detail previously [27]. To measure concentrations, internal standards were added to the samples. The serum was deproteinized on ice with dry 5-sulfosalicylic acid. Free amino acids were purified using cation exchange AG 50W-X8 resin (mesh size: 100–200, ionic form: hydrogen (Bio-Rad Laboratories, Hercules, CA, USA)) columns. The free amino acids were converted to their tert-butyl dimethylsilyl (MTBSTFA) derivative before analysis by GC-MS. The amino acid concentrations were determined using electron impact ionization by monitoring ions at mass/charge (m/z) 336 and 346 for unlabelled phenylalanine and internal standards, respectively. The serum phenylalanine ^13^C enrichments were determined using selective ion monitoring at m/z 336 and 342 for unlabelled and labelled phenylalanine, respectively. Standard regression curves were applied from a series of known standard enrichment values against the measured values to assess the linearity of the mass spectrometer and to account for any isotope fractionation that may have occurred during the analysis.

### Tissue analyses

As described in detail previously [27], all tissues were freeze-dried, weighed and crushed. Subsequently, samples were homogenized in ice-cold 2% perchloric acid (PCA) using ultrasonic disintegration (Soniprep; MSE, London, UK). Samples were incubated on ice for 10 min. Following centrifugation, the supernatant was collected for determination of L-[ring-^13^C_6_]-Phenylalanine enrichments in the tissue free amino acid pool using GC-MS analysis. Therefore, the supernatant was processed in the same manner as the serum samples. The tissue protein pellets were washed three times with 1.5 mL of ice-cold 2% PCA and hydrolysed in 3 mL of 6 M HCl overnight at 120°C. The free amino acids were then dissolved in 50% acetic acid solution and passed over cation exchange AG 50W-X8 resin (mesh size: 100–200, ionic form: hydrogen (Bio-Rad Laboratories, Hercules, CA, USA)) columns. The amino acids were eluted with 2 M NH_4_OH and dried under a continuous N_2_-stream for 48 h for measurement of L-[ring-^13^C_6_]-Phenylalanine enrichment in tissue protein. To determine the L-[ring-^13^C_6_]-Phenylalanine enrichment of tissue protein, the purified amino acids were derivatized into their N(O,S)-ethoxycarbonyl ethyl ester derivatives with ethyl chloroformate (ECF). The derivatives were then measured by GC-combustion-isotope ratio MS (GC-IRMS; MAT 253; Thermo-Scientific, Bremen, Germany) using an Agilent J&W DB-17MS (60 m) GC-column (Agilent Technologies, Santa Clara, CA, USA), and monitoring of ion masses 44, 45, and 46. Standard regression curves were applied to assess the linearity of the mass spectrometer and to control for the loss of tracer.

### Amino acid concentrations

Quantification of amino acids in the different tissues was performed using ultra-performance liquid chromatograph mass spectrometry (UPLC-MS; ACQUITY UPLC H-Class with QDa; Waters, Saint-Quentin, France), as described in detail previously [27]. At least 5 mg of freeze-dried tissue was hydrolysed in 3 mL of 6 M HCl for 12 h at 120°C and dried under a continuous N_2_-stream. 5 mL of 0.1 M HCl was used to reconstitute the hydrolysates after which 50 μL of each protein hydrolysate was deproteinized using 100 μL of 10% SSA with 50 μM of MSK-A2 internal standard (Cambridge Isotope Laboratories, Massachusetts, USA). Subsequently, 50 μL of ultra-pure demineralized water was added and samples were centrifuged. After centrifugation, 10 μL of supernatant was added to 70 μL of Borate reaction buffer (Waters, Saint-Quentin, France). In addition, 20 μL of AccQ-Tag derivatizing reagent solution (Waters, Saint-Quentin, France) was added after which the solution was heated to 55°C for 10 min. Of this 100 μL derivative 1 μL was injected and measured using UPLC-MS.

### Protein identification

As described in detail previously [27], tissue samples were homogenized in 50 mM ammonium bicarbonate and 5 M urea buffer, freeze-dried in three cycles, vortexed for 1 min and centrifuged at 20000*g* for 30 min at 10°C. The supernatant was collected and stored at -80°C until further analysis. Protein concentrations were determined with the Protein Assay Kit (Bio-Rad, Veenendaal, the Netherlands). Subsequently, a total of 75 μg protein in 50 μL 50 mM ammonium bicarbonate with 5 M urea was used for further analysis. 5 μL of DTT solution (20 mM final) was added and incubated at room temperature for 45 min. Proteins were alkylated by adding 6 μL of IAA solution (40 mM final) and incubated at room temperature for 45 min in darkness. Alkylation was stopped by adding 10 μL DTT solution (to consume any unreacted IAA) and incubation at room temperature for 45 min. Subsequently, 3 μg trypsin/lysC was added to the protein and incubated at 37°C for 2 h. 200 μL of 50 mM ammonium bicarbonate was added to dilute the urea concentration and the solution was further incubated at 37°C for 18 h. The digestion mixture was centrifuged at 2500*g* for 5 min and the supernatant was collected. The digestion mixture was fourfold diluted for the use of LC-MS/MS analysis. LC-MS/MS was performed using a nanoflow HPLC instrument (Dionex ultimate 3000) coupled on-line to a Q Exactive (Thermo Scientific) with a nano-electrospray Flex ion source (Proxeon). The digest/peptide mixture was loaded onto a C18-reversed phase column (Thermo Scientific Acclaim PepMap C18 column, 75-μm inner diameter x 15 cm, 2-μm particle size). Peptides were separated with a 90 min linear gradient of 4–45% buffer (80% acetonitrile and 0.08% formic acid) at a flow rate of 300 nL/min. Proteins were identified using Proteome Discoverer v2.1 Sequest HT search engine (Thermo Scientific). The false discovery rate (FDR) was set to 0.01 for proteins and peptides.

### Calculations

Tissue protein FSRs were calculated using the standard precursor-product equation:

$$FSR(\%/h)=\frac{E_{p2}-E_{p1}}{E_{precursor}\times t}\times 100\%$$

E_p2_ and E_p1_ are the protein-bound enrichments measured in the tissue samples collected during surgery and serum protein at *t* = -150 min (before the start of the tracer infusion), respectively (Fig 1) [28]. E_precursor_ is the average serum free L-[ring-^13^C_6_]-Phenylalanine or tissue free L-[ring-^13^C_6_]-Phenylalanine enrichments and *t* indicates the tracer incorporation time (measured from the start of the tracer infusion until excision of each specific tissue sample).

### Statistics

All data are expressed as means±SEM. Paired *t* tests were used to compare intracellular free and tissue protein bound L-[ring-^13^C_6_]-Phenylalanine enrichments between *vastus lateralis* muscle (as the reference tissue) and each of the different musculoskeletal tissues. Likewise, fractional synthesis rate of each of the different musculoskeletal tissues was compared with *vastus lateralis* muscle tissue fractional synthesis rates using paired *t* tests. For these comparisons, the 95% confidence interval (95% CI) of the difference, and the effect size (Cohen’s *d*) were calculated. No statistical analyses were performed between the different musculoskeletal tissues. Missing data was accounted for using pairwise deletion. Due to the exploratory nature of the experiment, multiplicity adjustments were not performed. For all analyses, significance was set at *P*<0.05. All calculations were performed using SPSS (version 23.0, IBM Corp., Armonk, NY, USA).

## Results

### Serum enrichments

**Fig 2:**
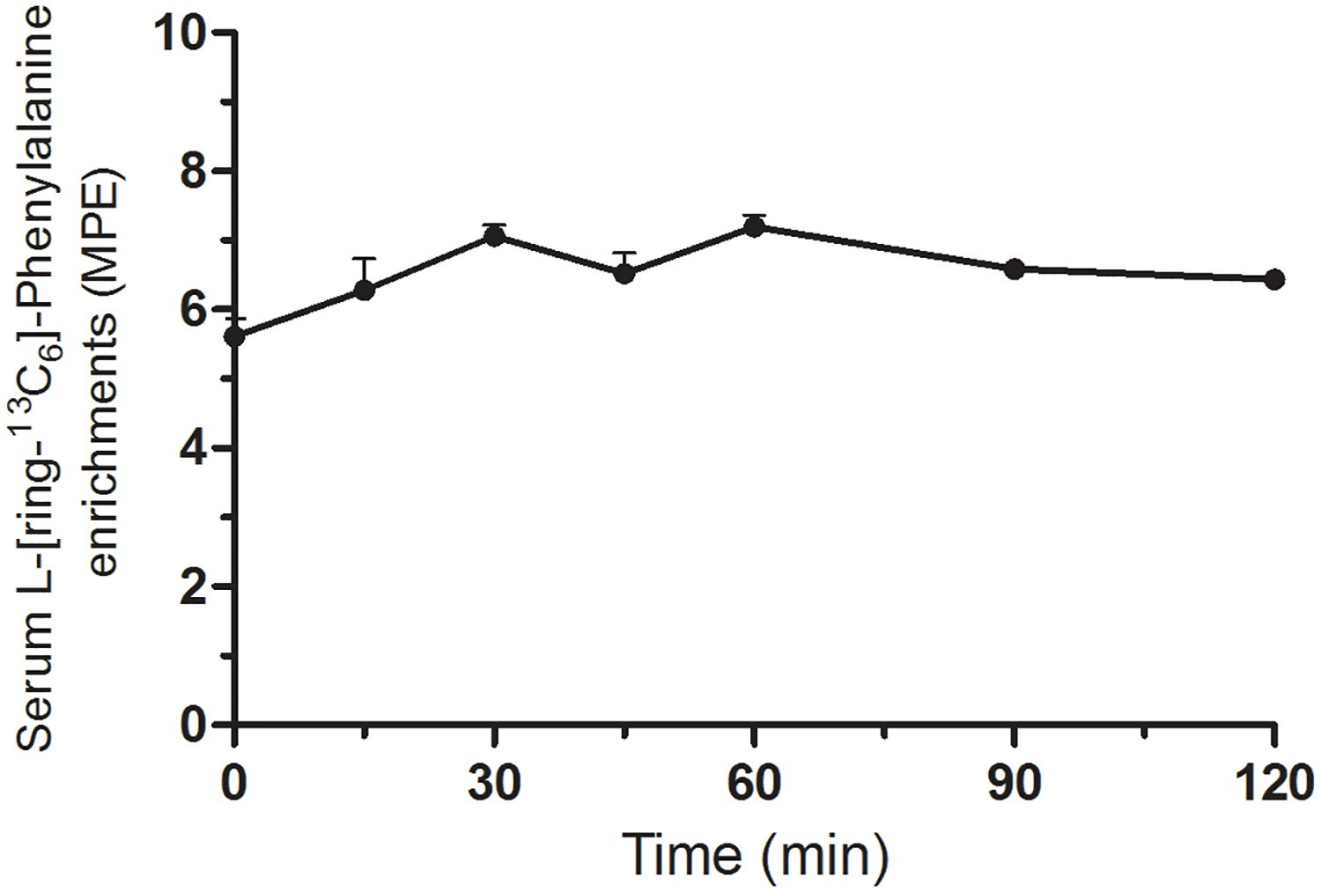
Serum L-[ring-^13^C_6_]-Phenylalanine enrichments. Serum L-[ring-^13^C_6_]-Phenylalanine enrichments are expressed as mole percent excess (MPE). *t* = 0 min represents the start of the surgical procedure. Values represent means+SEM. Serum L-[ring-^13^C_6_]-Phenylalanine enrichments did not change significantly throughout the experiments.

As shown in Fig 2, serum L-[ring-^13^C_6_]-Phenylalanine enrichments did not change significantly throughout the infusion period, despite the surgical setting of the experiment. Throughout the surgical procedure, serum L-[ring-^13^C_6_]-Phenylalanine enrichments averaged 6.53±0.20 MPE.

### Tissue free and protein bound enrichments

Tissue free L-[ring-^13^C_6_]-Phenylalanine enrichments in skeletal muscle tissue averaged 4.74±0.39 MPE. The majority of musculoskeletal tissues did not significantly differ in their tissue free L-[ring-^13^C_6_]-Phenylalanine enrichments when compared to skeletal muscle tissue, except for femoral bone, tibial bone, and Hoffa’s fat pad which all possessed lower tissue free L-[ring-^13^C_6_]-Phenylalanine enrichments (4.14±0.39, 4.19±0.35, and 4.32±0.41 MPE, respectively; *P*< 0.05; Table 1).

**Table 1:** Protein bound and tissue free L-[ring-^13^C_6_]-Phenylalanine enrichments.

Tissue	L-[ring-^13^C_6_]-Phenylalanine enrichment (MPE)	
	Protein bound	Tissue free
*Vastus lateralis*	0.010±0.002	4.74±0.39
Patellar bone	0.005±0.002	4.55±0.38
Femoral bone	0.006±0.002	4.14±0.39*
Tibial bone	0.006±0.002	4.19±0.35*
Notch	0.008±0.001*	4.72±0.47
Trochlea	0.007±0.002	4.59±0.48
Cartilage	0.009±0.002	4.86±0.35
Medial meniscus	0.009±0.002	5.19±0.31
Lateral meniscus	0.009±0.002	5.00±0.36
Patellar tendon	0.013±0.003	4.46±0.38
Anterior cruciate ligament	0.015±0.004	5.13±0.51
Posterior cruciate ligament	0.008±0.002	5.03±0.61
Hoffa’s fat pad	0.023±0.007	4.32±0.41*
Synovium	0.029±0.008	5.21±0.66

Values represent means±SEM. The number of pairs included in each comparison for both protein bound and tissue free L-[ring-^13^C_6_]-Phenylalanine enrichments is *n* = 6, except for tibial bone, trochlea, notch, and patellar bone tissue (all *n* = 5).

* significantly different from *vastus lateralis* muscle, *P*<0.01.

Protein bound L-[ring-^13^C_6_]-Phenylalanine enrichments in skeletal muscle tissue averaged 0.010±0.002 MPE. The highest enrichment levels were found in synovium and Hoffa’s fat pad (0.029±0.008 and 0.023±0.007 MPE, respectively), whereas the lowest L-[ring-^13^C_6_]-Phenylalanine enrichments were observed in patellar bone (0.005±0.002 MPE). The various musculoskeletal tissues did not significantly differ in their observed L-[ring-^13^C_6_]-Phenylalanine enrichments when compared to skeletal muscle, except for intercondylar notch bone tissue (i.e. the deep groove or notch between the two femoral condyles) that did have a lower L-[ring-^13^C_6_]-Phenylalanine enrichment level (0.010±0.002 vs 0.008±0.001, respectively; *P*<0.01; Table 1).

### Tissue protein synthesis rates

**Fig 3:**
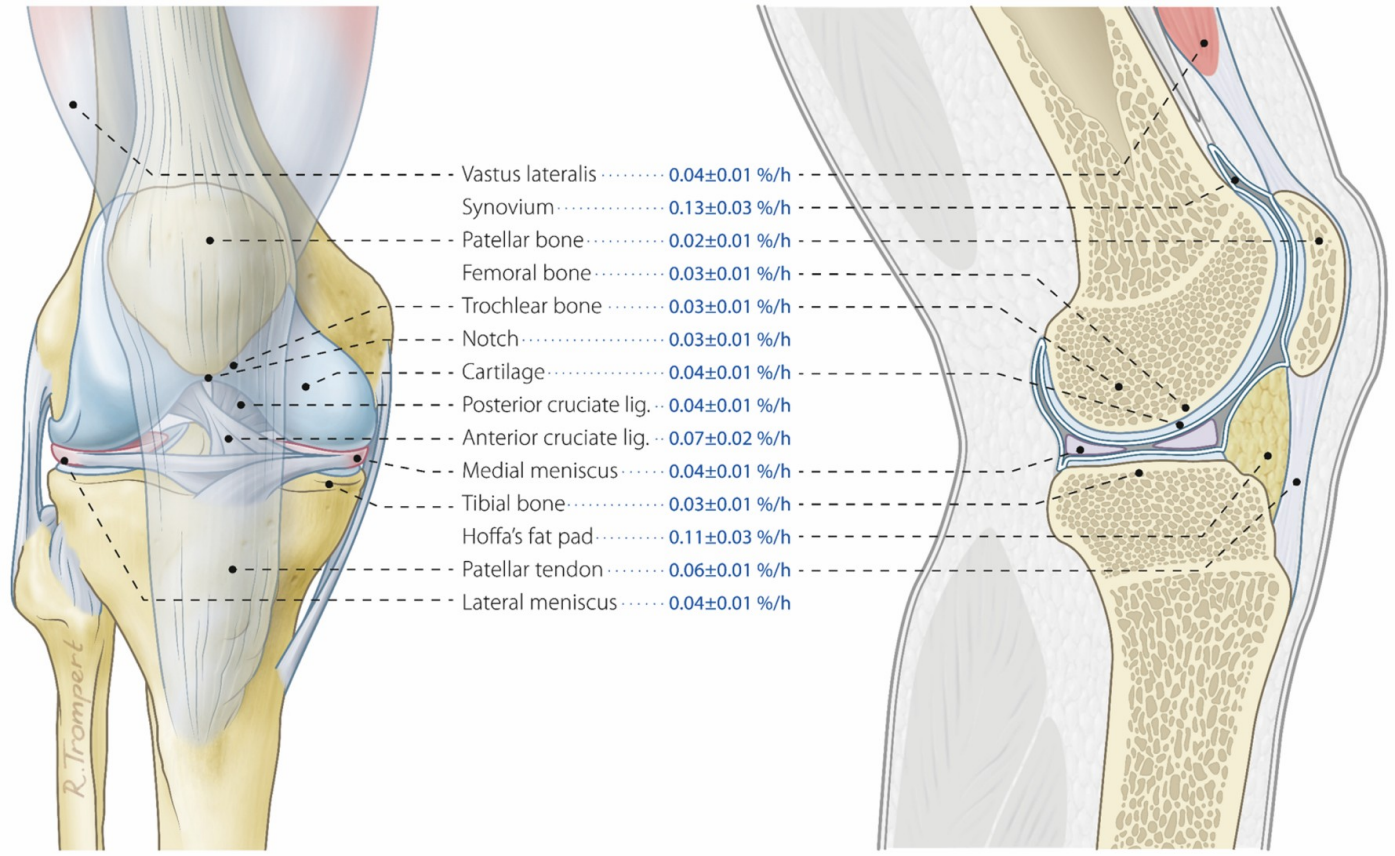
Anatomical illustration of musculoskeletal tissues and corresponding tissue protein synthesis rates. Tissue protein synthesis rates (FSR) based on incorporation of L-[ring-^13^C_6_]-Phenylalanine in human musculoskeletal tissue protein with serum L-[ring-^13^C_6_]-Phenylalanine enrichments used as precursor pool. Values represent means±SEM.

**Fig 4:**
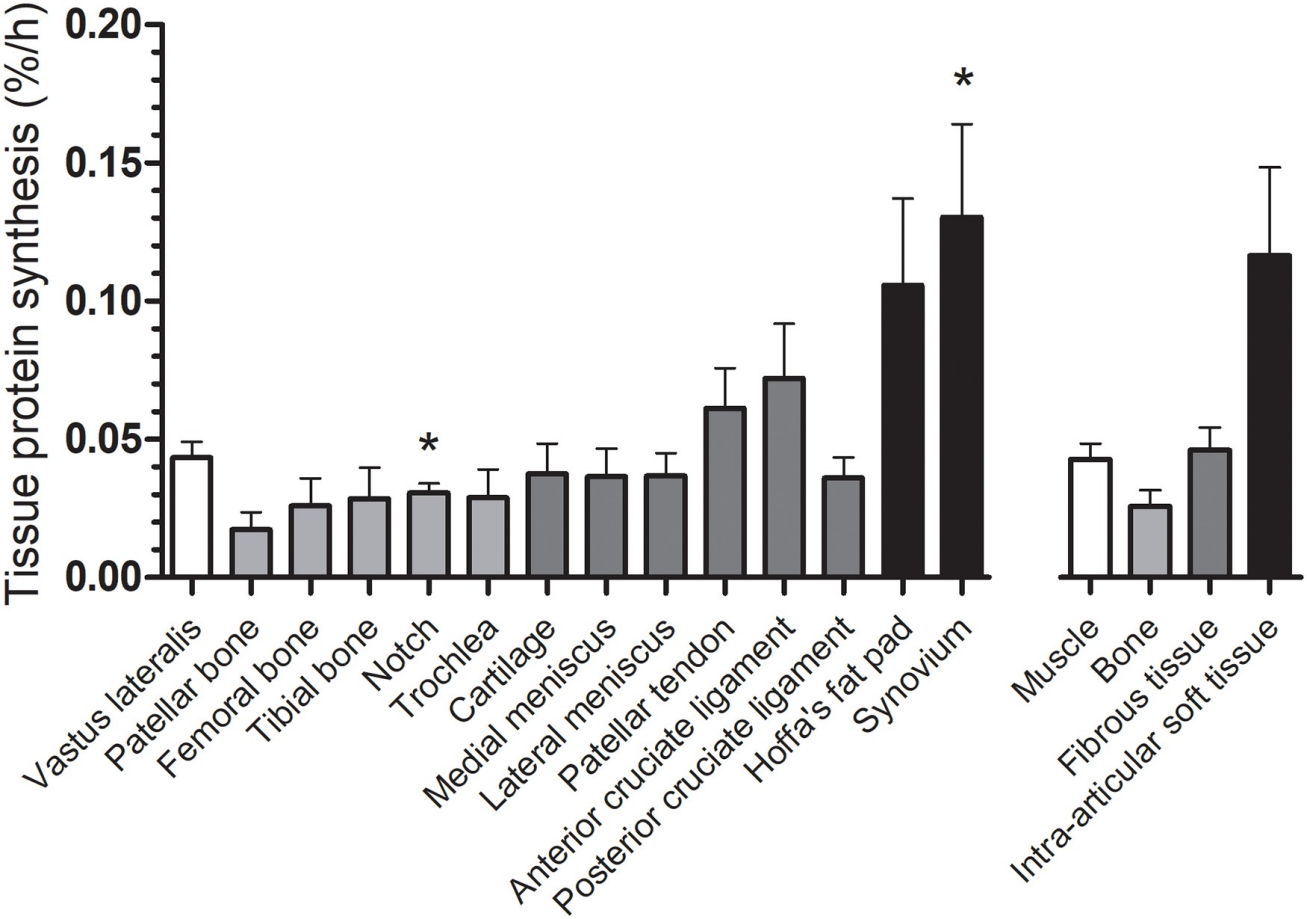
Musculoskeletal tissue protein synthesis rates. Fractional tissue protein synthesis rates (FSR) based on incorporation of L-[ring-^13^C_6_]-Phenylalanine in human musculoskeletal tissue protein with serum L-[ring-^13^C_6_]-Phenylalanine enrichments used as precursor pool. Values represent means+SEM. The number of pairs included in each comparison for both protein bound and tissue free L-[ring-^13^C_6_]-Phenylalanine enrichments is *n* = 6, except for tibial bone, trochlea, notch, and patellar bone tissue (all *n* = 5). The right-hand section x-axis represents averaged individual fractional synthesis rates per tissue class. * Significantly different from *vastus lateralis* muscle, *P*<0.05.

Tissue-specific protein synthesis rates, using serum L-[ring-^13^C_6_]-Phenylalanine enrichments as the precursor pool, are shown in Figs 3 and 4. In line with previous data, basal protein synthesis rates averaged 0.04±0.01%/h in skeletal muscle tissue. Synovium protein synthesis rates were significantly higher when compared to skeletal muscle protein synthesis rates (0.13±0.03%/h; *P*<0.05), whereas intercondylar notch bone tissue protein synthesis rates were significantly lower when compared to muscle tissue protein synthesis rates (0.03±0.01%/h; *P*<0.01). Fractional synthesis rates of other musculoskeletal tissues varied between 0.02 and 0.11%/h and did not significantly differ from skeletal muscle tissue. Please see Table 2 for the mean±SD of the difference, 95% CI of the difference, effect size (Cohen’s *d*), and *P*-value for the comparison of each musculoskeletal tissue with *vastus lateralis* muscle tissue protein synthesis rates. Similar tissue-specific protein synthesis rates were observed when using tissue free precursor enrichments, though significant differences were no longer present (S1 Fig).

**Table 2:** Musculoskeletal tissue protein synthesis rates compared to *vastus lateralis* protein synthesis rates.

Tissue	Mean±SD of the difference with *vastus lateralis* muscle (%/h)	95% CI of the difference (%/h)	Effect size (Cohen’s *d*)	*P*-value
Patellar bone	-0.02±0.02	-0.05–0.00	-1.07	0.08
Femoral bone	-0.02±0.03	-0.04–0.01	-0.67	0.16
Tibial bone	-0.02±0.03	-0.06–0.02	-0.62	0.24
Notch	-0.02±0.01	-0.03 –-0.01	-2.47	0.01
Trochlea	-0.02±0.02	-0.05–0.01	-0.83	0.14
Cartilage	-0.01±0.03	-0.03–0.02	-0.22	0.61
Medial meniscus	-0.01±0.03	-0.03–0.02	-0.26	0.56
Lateral meniscus	-0.01±0.03	-0.03–0.02	-0.25	0.57
Patellar tendon	0.02±0.03	-0.02–0.05	0.55	0.24
Anterior cruciate ligament	0.03±0.05	-0.03–0.08	0.54	0.24
Posterior cruciate ligament	-0.01±0.02	-0.03–0.01	-0.35	0.43
Hoffa’s fat pad	0.06±0.08	-0.02–0.14	0.80	0.11
Synovium	0.09±0.08	0.00–0.17	1.05	0.05

Values represent means±SEM. The number of pairs included in each comparison is *n* = 6, except for tibial bone, trochlea, notch, and patellar bone tissue (all *n* = 5).

### Tissue protein content and amino acid composition

Tissue protein contents ranged between 16 and 98% of the raw (dry) material (S1 Table). Protein contents of skeletal muscle averaged 74% and were higher when compared to the various bone tissue samples, which ranged between 16 and 31% of dry tissue weight. Compared to skeletal muscle tissue, protein contents of tendon, ligaments, cartilage, and menisci were higher and ranged between 79 and 98% of dry tissue weight. Protein content of synovium and Hoffa’s fat pad were lower compared to skeletal muscle tissue protein content (23±7 and 29±19% of dry tissue weight, respectively).

**Fig 5:**
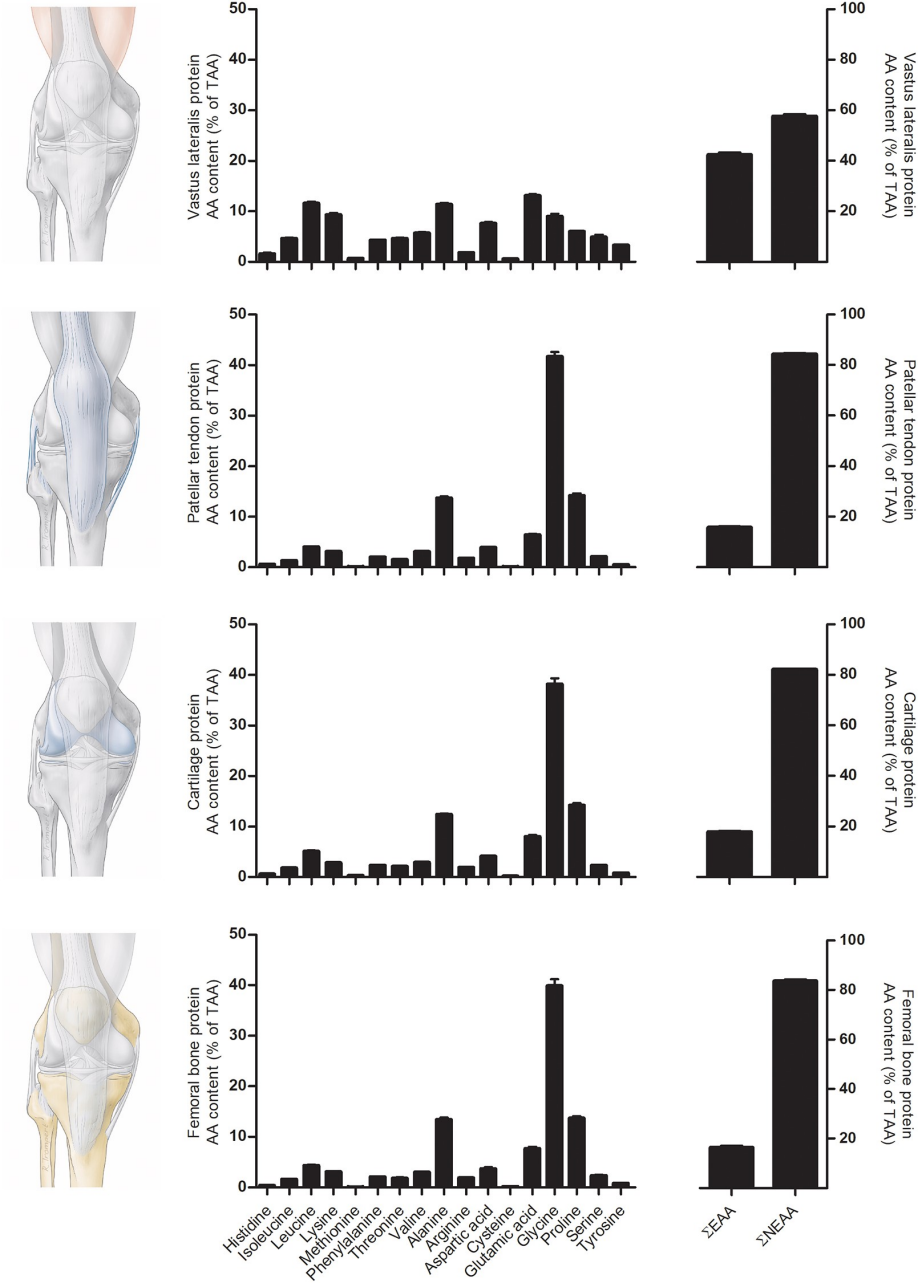
Amino acid content of muscle, tendon, cartilage, and bone tissue. Amino acid content is presented in % of total AA content. Note: Tryptophan, Asparagine, and Glutamine were not measured. ƩEAA, sum of all essential amino acids; ƩNEAA, sum of all non-essential amino acids.

Essential amino acid contents of all musculoskeletal tissues ranged between 15 and 25% of total amino acid content and were considerably lower when compared to skeletal muscle tissue (43% of total amino acids). Non-essential amino acid contents of the different musculoskeletal tissues ranged between 75 and 85% of total amino acids, were substantially higher compared to skeletal muscle (57% of total amino acids), and were mainly attributed to the high alanine, glycine, and proline contents (S1 Table). Amino acid profiles of tendon, cartilage, and bone differed substantially from skeletal muscle, with glycine contents as high as 42% of total amino acid content in patellar tendon, 39% in cartilage, and 40% in femoral bone tissue and as low as 9% in skeletal muscle tissue. In addition, proline contents appeared to be higher in all musculoskeletal tissues as well, with 14% of total amino acid content in patellar tendon and cartilage, 13.5% in femoral bone tissue, and 6% in skeletal muscle tissue. An overview of amino acid profile and amino acid composition, i.e. essential vs non-essential amino acid ratios, is provided in Fig 5.

### Protein identification

Supplemental S2 Table provides a list of all identified proteins and their corresponding estimated abundances. In total 1374 different proteins have been identified in the different musculoskeletal tissues of one subject (*n* = 1).

## Discussion

The current study provides insight into tissue protein metabolism of a wide variety of musculoskeletal tissues *in vivo* in humans. Using stable isotope methodology, we showed that average basal protein synthesis rates of various musculoskeletal tissues are within the same range of skeletal muscle protein synthesis rates, with fractional muscle, tendon, bone, cartilage, ligament, menisci, fat, and synovium protein synthesis rates ranging between 0.02 and 0.13% per hour *in vivo* in humans.

Skeletal muscle protein synthesis rates observed in the present study averaged 0.04±0.01%/h (Fig 3). These rates are similar to muscle protein synthesis rates assessed previously in an overnight fasted state in a wide variety of subjects studied in our lab [1, 29–34] as well as in many other laboratories [3, 35–37]. With protein synthesis rates ranging between 1–2% per 24 h, skeletal muscle tissue shows extensive remodeling within a matter of weeks to months [3]. These protein synthesis rates allow skeletal muscle tissue to adapt to changes in habitual use, with muscle hypertrophy following increased levels of physical activity [38–40] or muscle atrophy developing during periods of reduced physical activity or disuse [41–43]. Though a certain level of plasticity has been well established for skeletal muscle tissue, there is less data on *in vivo* tissue protein synthesis rates of most other musculoskeletal tissues in humans.

Tendon and ligaments play an important role in the force-transmitting function of the musculoskeletal system [18, 44]. Tendon protein synthesis rates in this study averaged 0.06±0.01%/h (Fig 3), which is in line with previous literature describing patellar tendon protein synthesis rates ranging between 0.01 and 0.07%/h [4–13, 15]. These data suggest that tendon tissue possesses similar protein synthesis characteristics as skeletal muscle tissue and may, therefore, also express some level of plasticity to external stimuli. In agreement, exercise has been reported to increase tendon protein synthesis rates [9, 13]. In addition to tendon tissue, total knee arthroplasty provided us with the unique opportunity to sample both anterior and posterior cruciate ligaments. With fractional synthetic rates averaging 0.07±0.02 and 0.04±0.01%/h for the anterior and posterior cruciate ligaments, respectively, we observed that both cruciate ligaments turn over at similar rates compared to skeletal muscle tissue (Fig 3). Interestingly, protein synthesis rates of patellar tendon tissue appeared to be similar to anterior cruciate ligament protein synthesis rates. Though not statistically tested, from a clinical perspective this may be relevant since patellar tendon tissue is often used in the surgical reconstruction of anterior cruciate ligament injuries [45]. In addition, protein synthesis rates of the anterior cruciate ligament tended to be higher compared to the posterior cruciate ligament (Fig 4). Whether this difference in protein synthesis rates between cruciate ligaments is reflective of differences in incidence of injuries [46] or the tissue’s capacity to repair, remains to be established.

Bone tissue quality is determined by multiple mechanical properties such as elasticity, resistance to bending, and toughness on impact [47, 48]. Though bone tissue has always been considered to possess limited remodeling capacity, few studies have actually investigated this. Previous studies have provided semi-quantitative estimates on adult bone remodeling of 3–25% per year [49], and on bone calcium turnover of ~8–15% per year [50, 51]. Here, we assessed bone tissue protein synthesis rates directly by measuring the incorporation of infused L-[ring-^13^C_6_]-Phenylalanine in the bone tissue protein pool of a variety of bone tissue samples. Bone tissue protein synthesis rates ranged between 0.02 and 0.03%/h (Fig 3). These data indicate that bone tissue may possess a much greater remodeling capacity than previously assumed. Though in this study most of the bone tissue samples showed protein synthesis rates similar to skeletal muscle tissue, much higher bone collagen synthesis rates (0.06±0.01%/h) have been observed previously by Babraj *et al*. using stable isotope methodology [52]. This discrepancy in synthesis rates may be caused by the type of protein studied. Babraj *et al*. [52] have measured bone collagen synthesis rates, whereas we have assessed mixed bone tissue protein synthesis rates. Obviously, protein synthetic rates may differ substantially between different proteins and protein fractions.

To our knowledge, no previous study has applied stable isotope methodology to assess fractional protein synthesis rates of cartilage and menisci *in vivo* in humans. These structures have always been suggested to turn over slowly. Especially human cartilage has been considered an essentially permanent structure with little to no ability to remodel after a certain age [53]. Our data, however, show that cartilage tissue protein synthesis rates average 0.04±0.01%/h. In addition, we observed that both medial and lateral menisci have a protein synthesis rate of 0.04±0.01 and 0.04±0.01%/h, respectively as well (Fig 3). Since cartilage tissue is avascular [25] and menisci tissue’s vascularization differs substantially within the tissue [21], tissue-specific protein synthesis rates were also calculated based on tissue free L-[ring-^13^C_6_]-Phenylalanine enrichments. However, a similar pattern of tissue-specific protein synthesis rates was observed when using tissue free precursor enrichments.

Our assessment of musculoskeletal tissue protein synthesis rates represents the integrated synthesis rates of all available proteins in the musculoskeletal tissues that were sampled. However, different proteins or protein fractions within a tissue likely possess different turnover rates. Indeed, others have shown that protein turnover of the collagen matrix of human articular cartilage is negligible whereas turnover rates of the cartilage glycosaminoglycan matrix are substantially higher [53]. Hence, it seems that differences between proteins and protein fractions within different musculoskeletal tissues may be important in interpreting these data. To obtain more insight into the contribution of individual proteins and/or protein fractions to the mixed tissue protein synthesis rates we report here, we additionally applied liquid chromatography/mass spectrometry (LC-MS/MS) in all tissue samples of a single subject to identify the proteins present and their (semi-quantitative) abundance in the tissues. Supplementary S2 Table provides a list of 1374 identified proteins and their estimated abundances in each of the musculoskeletal tissues. Though such data provide more in-depth insight, they do not show which proteins or protein fractions are synthesized more or less rapidly. The combination of contemporary stable isotope methodology and proteomics analyses methods do not yet allow us to detect fractional protein synthesis rates at the level of individual proteins in tissues *in vivo* in humans. Limitations are present in the ability to identify all proteins present, their (relative) abundances in the tissues, as well as their degree of label enrichments.

To obtain some insight into the differences in amino acid composition between the different tissues, we applied ultra-performance liquid chromatography mass spectrometry (UPLC-MS) to assess tissue-specific amino acid composition of all musculoskeletal tissues (S1 Table). Fig 5 presents a selection of 4 different musculoskeletal tissues and their amino acid composition. Essential amino acid contents of patellar tendon, cartilage, and femoral bone are much lower when compared to skeletal muscle tissue, whereas the amino acids glycine and proline are substantially more abundant in patellar tendon, cartilage, and femoral bone when compared to skeletal muscle tissue. Proline and glycine are both amino acids known to be important in musculoskeletal collagen metabolism [54–56]. Future research should evaluate the impact of such differences in amino acid content on basal and, more importantly, post-prandial tissue protein synthesis rates under various conditions.

Apart from musculoskeletal tissues that allow for adequate movement and power transfer, the human knee joint also contains metabolically active intra-articular soft tissues such as Hoffa’s fat pad and synovium. Hoffa’s fat pad is known to share morphological similarities with subcutaneous fat [57], possess an abundant peripheral anastomotic blood supply [58], and has been suggested to play a modulatory role in the inflammatory pathways in osteoarthritis [59]. Synovium tissue lines the inner surface of the knee joint and primarily produces synovial fluid to lubricate the joint. However, it is also known to become inflamed in patients with different stages of knee osteoarthritis [60]. In the present study Hoffa’s fat pad and synovium tissue protein synthesis rates averaged 0.11±0.03 and 0.13±0.03%/h, respectively (Fig 3), which are substantially higher when compared to skeletal muscle tissue protein synthesis rates (Fig 4). These higher tissue protein synthesis rates of Hoffa’s fat pad and synovium tissue might be reflective of the metabolic active nature of these tissues or their suggested role in disease dependent pathways. For all tissues we ensured that only visually unaffected parts of the tissue were sampled, thereby ensuring that we were sampling only healthy tissue. However, the potential influence of disease dependent processes on our measurement of tissue protein synthesis rates in Hoffa’s fat pad and synovium should not be ignored. Nevertheless, these observations for the first time show tissue protein synthesis rates of intra-articular soft tissues when compared to other musculoskeletal tissues *in vivo* in humans.

From a research perspective, the present study provides interesting data on protein metabolism of a wide variety of musculoskeletal tissues of the human knee. Whereas protein synthesis rates have been assessed in some of the referred tissues, no study has directly assessed *in vivo* protein synthesis rates of all of these musculoskeletal tissues in a single study in humans. Knowledge of basal musculoskeletal tissue protein synthesis rates enables us to further explore the capacity of these tissues to regenerate. Skeletal muscle plasticity is well appreciated since muscle tissue has shown to be highly responsive to both anabolic and catabolic stimuli. To assess whether other musculoskeletal tissues are capable of displaying some degree of plasticity, more work is required to address the impact of various factors on tissue protein synthesis rates. It has previously been observed that gelatin supplementation can stimulate collagen synthesis following exercise [55], and collagen hydrolysate supplementation has been reported to increase collagen content in the knee of osteoarthritis patients [61], and may decrease knee pain in athletes with activity-related joint pain [62]. From a clinical perspective this is more than interesting, because identifying which specific proteins or protein fractions are responsive to external stimuli may enable us to develop more effective therapies in treating and/or preventing injuries.

In conclusion, basal fractional muscle, tendon, bone, cartilage, ligament and menisci protein synthesis rates range between 0.02 and 0.13% per hour *in vivo* in humans. Fractional tissue protein synthesis rates of tendon, bone, cartilage, ligament and menisci do not differ substantially from muscle tissue protein synthesis rates, suggesting that these musculoskeletal tissues may express a greater level of tissue plasticity than generally believed.

## Supporting information

S1 Fig Musculoskeletal tissue protein synthesis rates. Fractional tissue protein synthesis rates (FSR) based on incorporation of L-[ring-^13^C_6_]-Phenylalanine in human musculoskeletal tissue protein with tissue free L-[ring-^13^C_6_]-Phenylalanine enrichments used as precursor pool. Values represent means+SEM. The number of pairs included in each comparison for both protein bound and tissue free L-[ring-^13^C_6_]-Phenylalanine enrichments is *n* = 6, except for tibial bone, trochlea, notch, and patellar bone tissue (all *n* = 5). * Significantly different from *vastus lateralis* muscle, *P*<0.05.
(TIF)

S1 Table Protein and amino acid content of various musculoskeletal tissues. Protein content is presented in % of raw material based on the determined nitrogen content multiplied by 6.25 as the standard conversion factor. Amino acid content is presented in % of total AA content. Note: Tryptophan, Asparagine, and Glutamine were not measured. ƩEAA, sum of all essential amino acids; ƩNEAA, sum of all non-essential amino acids. URL: https://osf.io/z7bgk/?view_only=9400d38f9c0749599a64cbf4e5682f91.
(DOCX)

S2 Table Protein identification. Protein identification and semi-quantitative analyses of relative abundances were performed by LC-MS/MS. URL: https://osf.io/z7bgk/?view_only=9400d38f9c0749599a64cbf4e5682f91.
(XLSX)

## Abbreviations

AA: amino acid
FSR: fractional synthesis rate
GC-IRMS: gas chromatography isotope ratio mass spectrometry
GC-MS: gas chromatography—mass spectrometry
LC-MS/MS: liquid chromatography—mass spectrometry
MPE: mole percent excess
PCA: perchloric acid
UPLC-MS: ultra-performance liquid chromatography mass spectrometry

## References

[1] Burd NA, Groen BB, Beelen M, Senden JM, Gijsen AP, van Loon LJ. The reliability of using the single-biopsy approach to assess basal muscle protein synthesis rates in vivo in humans. Metabolism. 2012;61(7):931–6. 10.1016/j.metabol.2011.11.004 . 22209666

[2] Rennie MJ, Smith K, Watt PW. Measurement of human tissue protein synthesis: an optimal approach. Am J Physiol. 1994;266(3 Pt 1):E298–307. 10.1152/ajpendo.1994.266.3.E298 . 8166250

[3] Waterlow JC. Protein turnover. Oxfordshire: CABI; 2006.

[4] Babraj JA, Cuthbertson DJ, Smith K, Langberg H, Miller B, Krogsgaard MR, et al Collagen synthesis in human musculoskeletal tissues and skin. Am J Physiol Endocrinol Metab. 2005;289(5):E864–9. 10.1152/ajpendo.00243.2005 . 15972270

[5] Dideriksen K, Sindby AK, Krogsgaard M, Schjerling P, Holm L, Langberg H. Effect of acute exercise on patella tendon protein synthesis and gene expression. Springerplus. 2013;2(1):109 10.1186/2193-1801-2-109 . 23586004 PMC3622742

[6] Hansen M, Boesen A, Holm L, Flyvbjerg A, Langberg H, Kjaer M. Local administration of insulin-like growth factor-I (IGF-I) stimulates tendon collagen synthesis in humans. Scand J Med Sci Sports. 2013;23(5):614–9. 10.1111/j.1600-0838.2011.01431.x . 22288768

[7] Hansen M, Miller BF, Holm L, Doessing S, Petersen SG, Skovgaard D, et al Effect of administration of oral contraceptives in vivo on collagen synthesis in tendon and muscle connective tissue in young women. J Appl Physiol (1985). 2009;106(4):1435–43. 10.1152/japplphysiol.90933.2008 . 18845777

[8] Miller BF, Hansen M, Olesen JL, Schwarz P, Babraj JA, Smith K, et al Tendon collagen synthesis at rest and after exercise in women. J Appl Physiol (1985). 2007;102(2):541–6. 10.1152/japplphysiol.00797.2006 . 16990502

[9] Miller BF, Olesen JL, Hansen M, Dossing S, Crameri RM, Welling RJ, et al Coordinated collagen and muscle protein synthesis in human patella tendon and quadriceps muscle after exercise. J Physiol. 2005;567(Pt 3):1021–33. 10.1113/jphysiol.2005.093690 . 16002437 PMC1474228

[10] Nielsen RH, Doessing S, Goto K, Holm L, Reitelseder S, Agergaard J, et al GH receptor blocker administration and muscle-tendon collagen synthesis in humans. Growth Horm IGF Res. 2011;21(3):140–5. 10.1016/j.ghir.2011.03.006 . 21498100

[11] Nielsen RH, Holm L, Jensen JK, Heinemeier KM, Remvig L, Kjaer M. Tendon protein synthesis rate in classic Ehlers-Danlos patients can be stimulated with insulin-like growth factor-I. J Appl Physiol (1985). 2014;117(7):694–8. 10.1152/japplphysiol.00157.2014 . 25103963

[12] Nielsen RH, Holm L, Malmgaard-Clausen NM, Reitelseder S, Heinemeier KM, Kjaer M. Increase in tendon protein synthesis in response to insulin-like growth factor-I is preserved in elderly men. J Appl Physiol (1985). 2014;116(1):42–6. 10.1152/japplphysiol.01084.2013 . 24265284

[13] Petersen SG, Miller BF, Hansen M, Kjaer M, Holm L. Exercise and NSAIDs: effect on muscle protein synthesis in patients with knee osteoarthritis. Med Sci Sports Exerc. 2011;43(3):425–31. 10.1249/MSS.0b013e3181f27375 . 20689451

[14] Dideriksen K, Boesen AP, Reitelseder S, Couppe C, Svensson R, Schjerling P, et al Tendon collagen synthesis declines with immobilization in elderly humans: no effect of anti-inflammatory medication. J Appl Physiol (1985). 2017;122(2):273–82. 10.1152/japplphysiol.00809.2015 . 27932679

[15] Doessing S, Heinemeier KM, Holm L, Mackey AL, Schjerling P, Rennie M, et al Growth hormone stimulates the collagen synthesis in human tendon and skeletal muscle without affecting myofibrillar protein synthesis. J Physiol. 2010;588(Pt 2):341–51. 10.1113/jphysiol.2009.179325 . 19933753 PMC2821728

[16] Magnusson SP, Heinemeier KM, Kjaer M. Collagen Homeostasis and Metabolism. Adv Exp Med Biol. 2016;920:11–25. Epub 2016/08/19. 10.1007/978-3-319-33943-6_2 . 27535245

[17] Baar K. Minimizing Injury and Maximizing Return to Play: Lessons from Engineered Ligaments. Sports Med. 2017;47(Suppl 1):5–11. Epub 2017/03/24. 10.1007/s40279-017-0719-x . 28332110 PMC5371618

[18] Kjaer M. Role of extracellular matrix in adaptation of tendon and skeletal muscle to mechanical loading. Physiol Rev. 2004;84(2):649–98. 10.1152/physrev.00031.2003 . 15044685

[19] Kjaer M, Langberg H, Heinemeier K, Bayer ML, Hansen M, Holm L, et al From mechanical loading to collagen synthesis, structural changes and function in human tendon. Scand J Med Sci Sports. 2009;19(4):500–10. 10.1111/j.1600-0838.2009.00986.x . 19706001

[20] Bergstrom J. Percutaneous needle biopsy of skeletal muscle in physiological and clinical research. Scand J Clin Lab Invest. 1975;35(7):609–16. . 1108172

[21] Bryceland JK, Powell AJ, Nunn T. Knee Menisci. Cartilage. 2017;8(2):99–104. Epub 2017/03/28. 10.1177/1947603516654945 . 28345407 PMC5358830

[22] Marenzana M, Arnett TR. The Key Role of the Blood Supply to Bone. Bone Res. 2013;1(3):203–15. Epub 2013/09/01. 10.4248/BR201303001 . 26273504 PMC4472103

[23] Nemschak G, Pretterklieber ML. The Patellar Arterial Supply via the Infrapatellar Fat Pad (of Hoffa): A Combined Anatomical and Angiographical Analysis. Anat Res Int. 2012;2012:713838 Epub 2012/06/22. 10.1155/2012/713838 . 22720162 PMC3375036

[24] Smith MD. The normal synovium. Open Rheumatol J. 2011;5:100–6. Epub 2012/01/27. 10.2174/1874312901105010100 . 22279508 PMC3263506

[25] Sophia Fox AJ, Bedi A, Rodeo SA. The basic science of articular cartilage: structure, composition, and function. Sports Health. 2009;1(6):461–8. Epub 2009/11/01. 10.1177/1941738109350438 . 23015907 PMC3445147

[26] Travascio F, Jackson AR. The nutrition of the human meniscus: A computational analysis investigating the effect of vascular recession on tissue homeostasis. J Biomech. 2017;61:151–9. Epub 2017/08/06. 10.1016/j.jbiomech.2017.07.019 . 28778387

[27] Smeets JSJ, Horstman AMH, Schijns O, Dings JTA, Hoogland G, Gijsen AP, et al Brain tissue plasticity: protein synthesis rates of the human brain. Brain. 2018;141(4):1122–9. Epub 2018/02/13. 10.1093/brain/awy015 . 29432531

[28] Burd NA, West DW, Rerecich T, Prior T, Baker SK, Phillips SM. Validation of a single biopsy approach and bolus protein feeding to determine myofibrillar protein synthesis in stable isotope tracer studies in humans. Nutr Metab (Lond). 2011;8:15 Epub 2011/03/11. 10.1186/1743-7075-8-15 . 21388545 PMC3068071

[29] Gorissen SH, Horstman AM, Franssen R, Crombag JJ, Langer H, Bierau J, et al Ingestion of Wheat Protein Increases In Vivo Muscle Protein Synthesis Rates in Healthy Older Men in a Randomized Trial. J Nutr. 2016;146(9):1651–9. 10.3945/jn.116.231340 . 27440260

[30] Gorissen SH, Horstman AM, Franssen R, Kouw IW, Wall BT, Burd NA, et al Habituation to low or high protein intake does not modulate basal or postprandial muscle protein synthesis rates: a randomized trial. Am J Clin Nutr. 2017;105(2):332–42. 10.3945/ajcn.115.129924 . 27903518

[31] Hursel R, Martens EA, Gonnissen HK, Hamer HM, Senden JM, van Loon LJ, et al Prolonged Adaptation to a Low or High Protein Diet Does Not Modulate Basal Muscle Protein Synthesis Rates—A Substudy. PLoS One. 2015;10(9):e0137183 10.1371/journal.pone.0137183 . 26367529 PMC4569069

[32] Kouw IW, Gorissen SH, Burd NA, Cermak NM, Gijsen AP, van Kranenburg J, et al Postprandial Protein Handling Is Not Impaired in Type 2 Diabetes Patients When Compared With Normoglycemic Controls. J Clin Endocrinol Metab. 2015;100(8):3103–11. 10.1210/jc.2015-1234 . 26037513

[33] Kramer IF, Verdijk LB, Hamer HM, Verlaan S, Luiking YC, Kouw IW, et al Both basal and post-prandial muscle protein synthesis rates, following the ingestion of a leucine-enriched whey protein supplement, are not impaired in sarcopenic older males. Clin Nutr. 2016 10.1016/j.clnu.2016.09.023 . 27743615

[34] Wall BT, Gorissen SH, Pennings B, Koopman R, Groen BB, Verdijk LB, et al Aging Is Accompanied by a Blunted Muscle Protein Synthetic Response to Protein Ingestion. PLoS One. 2015;10(11):e0140903 10.1371/journal.pone.0140903 . 26536130 PMC4633096

[35] Volpi E, Sheffield-Moore M, Rasmussen BB, Wolfe RR. Basal muscle amino acid kinetics and protein synthesis in healthy young and older men. JAMA. 2001;286(10):1206–12. 10.1001/jama.286.10.1206 . 11559266 PMC3183815

[36] Yang Y, Breen L, Burd NA, Hector AJ, Churchward-Venne TA, Josse AR, et al Resistance exercise enhances myofibrillar protein synthesis with graded intakes of whey protein in older men. Br J Nutr. 2012;108(10):1780–8. 10.1017/S0007114511007422 . 22313809

[37] Yang Y, Churchward-Venne TA, Burd NA, Breen L, Tarnopolsky MA, Phillips SM. Myofibrillar protein synthesis following ingestion of soy protein isolate at rest and after resistance exercise in elderly men. Nutr Metab (Lond). 2012;9(1):57 10.1186/1743-7075-9-57 . 22698458 PMC3478988

[38] Cermak NM, Res PT, de Groot LC, Saris WH, van Loon LJ. Protein supplementation augments the adaptive response of skeletal muscle to resistance-type exercise training: a meta-analysis. Am J Clin Nutr. 2012;96(6):1454–64. 10.3945/ajcn.112.037556 . 23134885

[39] Chesley A, MacDougall JD, Tarnopolsky MA, Atkinson SA, Smith K. Changes in human muscle protein synthesis after resistance exercise. J Appl Physiol (1985). 1992;73(4):1383–8. 10.1152/jappl.1992.73.4.1383 . 1280254

[40] Phillips SM, Tipton KD, Aarsland A, Wolf SE, Wolfe RR. Mixed muscle protein synthesis and breakdown after resistance exercise in humans. Am J Physiol. 1997;273(1 Pt 1):E99–107. 10.1152/ajpendo.1997.273.1.E99 . 9252485

[41] Deitrick JE. The effect of immobilization on metabolic and physiological functions of normal men. Bull N Y Acad Med. 1948;24(6):364–75. . 18860463 PMC1871484

[42] Gibson JN, Halliday D, Morrison WL, Stoward PJ, Hornsby GA, Watt PW, et al Decrease in human quadriceps muscle protein turnover consequent upon leg immobilization. Clin Sci (Lond). 1987;72(4):503–9. 10.1042/cs0720503 . 2435445

[43] Ingemann-Hansen T, Halkjaer-Kristensen J. Computerized tomographic determination of human thigh components. The effects of immobilization in plaster and subsequent physical training. Scand J Rehabil Med. 1980;12(1):27–31. . 7384763

[44] Finni T, Komi PV, Lepola V. In vivo human triceps surae and quadriceps femoris muscle function in a squat jump and counter movement jump. Eur J Appl Physiol. 2000;83(4–5):416–26. 10.1007/s004210000289 . 11138584

[45] Shelbourne KD, Beck MB, Gray T. Anterior cruciate ligament reconstruction with contralateral autogenous patellar tendon graft: evaluation of donor site strength and subjective results. Am J Sports Med. 2015;43(3):648–53. Epub 2014/12/19. 10.1177/0363546514560877 . 25520302

[46] Majewski M, Susanne H, Klaus S. Epidemiology of athletic knee injuries: A 10-year study. Knee. 2006;13(3):184–8. Epub 2006/04/11. 10.1016/j.knee.2006.01.005 . 16603363

[47] Bailey AJ, Sims TJ, Ebbesen EN, Mansell JP, Thomsen JS, Mosekilde L. Age-related changes in the biochemical properties of human cancellous bone collagen: relationship to bone strength. Calcif Tissue Int. 1999;65(3):203–10. 10.1007/s002239900683 . 10441651

[48] Zioupos P. Ageing human bone: factors affecting its biomechanical properties and the role of collagen. J Biomater Appl. 2001;15(3):187–229. 10.1106/5JUJ-TFJ3-JVVA-3RJ0 . 11261600

[49] Mundy GR. Bone resorption and turnover in health and disease. Bone. 1987;8 Suppl 1:S9–16. . 3318891

[50] Abrams SA. Normal acquisition and loss of bone mass. Horm Res. 2003;60 Suppl 3:71–6. 10.1159/000074505 . 14671401

[51] Heaney RP, Abrams S, Dawson-Hughes B, Looker A, Marcus R, Matkovic V, et al Peak bone mass. Osteoporos Int. 2000;11(12):985–1009. 10.1007/s001980070020 . 11256898

[52] Babraj JA, Smith K, Cuthbertson DJ, Rickhuss P, Dorling JS, Rennie MJ. Human bone collagen synthesis is a rapid, nutritionally modulated process. J Bone Miner Res. 2005;20(6):930–7. 10.1359/JBMR.050201 . 15883632

[53] Heinemeier KM, Schjerling P, Heinemeier J, Moller MB, Krogsgaard MR, Grum-Schwensen T, et al Radiocarbon dating reveals minimal collagen turnover in both healthy and osteoarthritic human cartilage. Sci Transl Med. 2016;8(346):346ra90 10.1126/scitranslmed.aad8335 . 27384346

[54] Paxton JZ, Grover LM, Baar K. Engineering an in vitro model of a functional ligament from bone to bone. Tissue Eng Part A. 2010;16(11):3515–25. 10.1089/ten.TEA.2010.0039 . 20593972

[55] Shaw G, Lee-Barthel A, Ross ML, Wang B, Baar K. Vitamin C-enriched gelatin supplementation before intermittent activity augments collagen synthesis. Am J Clin Nutr. 2017;105(1):136–43. 10.3945/ajcn.116.138594 . 27852613 PMC5183725

[56] Vieira CP, De Oliveira LP, Da Re Guerra F, Dos Santos De Almeida M, Marcondes MC, Pimentel ER. Glycine improves biochemical and biomechanical properties following inflammation of the achilles tendon. Anat Rec (Hoboken). 2015;298(3):538–45. 10.1002/ar.23041 . 25156668

[57] Saddik D, McNally EG, Richardson M. MRI of Hoffa’s fat pad. Skeletal Radiol. 2004;33(8):433–44. 10.1007/s00256-003-0724-z . 15221217

[58] Kohn D, Deiler S, Rudert M. Arterial blood supply of the infrapatellar fat pad. Anatomy and clinical consequences. Arch Orthop Trauma Surg. 1995;114(2):72–5. 10.1007/bf00422828 . 7734236

[59] Clockaerts S, Bastiaansen-Jenniskens YM, Runhaar J, Van Osch GJ, Van Offel JF, Verhaar JA, et al The infrapatellar fat pad should be considered as an active osteoarthritic joint tissue: a narrative review. Osteoarthritis Cartilage. 2010;18(7):876–82. 10.1016/j.joca.2010.03.014 . 20417297

[60] Ene R, Sinescu RD, Ene P, Cirstoiu MM, Cirstoiu FC. Synovial inflammation in patients with different stages of knee osteoarthritis. Rom J Morphol Embryol. 2015;56(1):169–73. . 25826502

[61] McAlindon TE, Nuite M, Krishnan N, Ruthazer R, Price LL, Burstein D, et al Change in knee osteoarthritis cartilage detected by delayed gadolinium enhanced magnetic resonance imaging following treatment with collagen hydrolysate: a pilot randomized controlled trial. Osteoarthritis Cartilage. 2011;19(4):399–405. 10.1016/j.joca.2011.01.001 . 21251991

[62] Clark KL, Sebastianelli W, Flechsenhar KR, Aukermann DF, Meza F, Millard RL, et al 24-Week study on the use of collagen hydrolysate as a dietary supplement in athletes with activity-related joint pain. Curr Med Res Opin. 2008;24(5):1485–96. 10.1185/030079908X291967 . 18416885

