# Supplementary figures and images for: Protein synthesis rates of muscle, tendon, ligament, cartilage, and bone tissue in vivo in humans
Source: PLoS One. 2019 Nov 7;14(11):e0224745. doi: 10.1371/journal.pone.0224745 (PMC6837426; doi:10.1371/journal.pone.0224745)

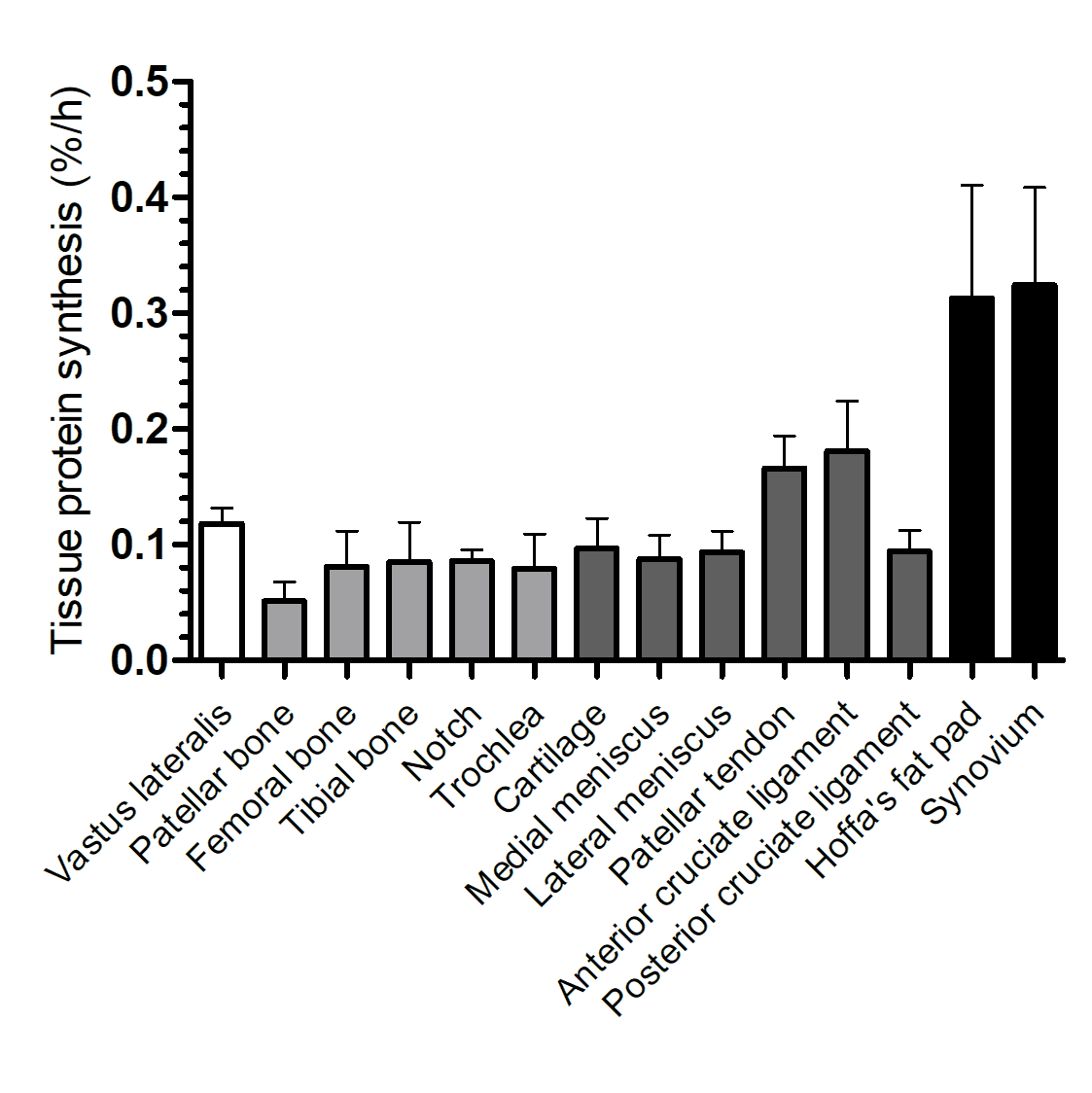

Supplement: S1 Fig — Fractional tissue protein synthesis rates (FSR) based on incorporation of L-[ring-13C6]-Phenylalanine in human musculoskeletal tissue protein with tissue free L-[ring-13C6]-Phenylalanine enrichments used as precursor pool. Values represent means+SEM. The number of pairs included in each comparison for both protein bound and tissue free L-[ring-13C6]-Phenylalanine enrichments is n = 6, except for tibial bone, trochlea, notch, and patellar bone tissue (all n = 5). * Significantly different from vastus lateralis muscle, P<0.05. (TIF) [file pone.0224745.s001.tif]
