# Supplementary material for: Protein synthesis rates of muscle, tendon, ligament, cartilage, and bone tissue in vivo in humans
Source: PLoS One. 2019 Nov 7;14(11):e0224745. doi: 10.1371/journal.pone.0224745 (PMC6837426; doi:10.1371/journal.pone.0224745)
Supplement: S1 Table — Protein content is presented in % of raw material based on the determined nitrogen content multiplied by 6.25 as the standard conversion factor. Amino acid content is presented in % of total AA content. Note: Tryptophan, Asparagine, and Glutamine were not measured. ƩEAA, sum of all essential amino acids; ƩNEAA, sum of all non-essential amino acids. URL: https://osf.io/z7bgk/?view_only=9400d38f9c0749599a64cbf4e5682f91. (DOCX) [file pone.0224745.s002.docx]

**Table S1**

|  | ***Vastus lateralis*** | **Patellar bone** | **Femoral bone** | **Tibial bone** | **Notch** | **Trochlea** | **Cartilage** | **Medial meniscus** | **Lateral meniscus** | **Patellar tendon** | **Anterior cruciate ligament** | **Posterior cruciate ligament** | **Hoffa’s fat pad** | **Synovium** |
| --- | --- | --- | --- | --- | --- | --- | --- | --- | --- | --- | --- | --- | --- | --- |
| **Total nitrogen content (% dry weight)** | 12±1 | 5±0 | 4±1 | 5±1 | 2±0 | 3±0 | 14±0 | 16±0 | 14±1 | 13±1 | 15±1 | 13±2 | 5±3 | 4±1 |
| **Total protein content (% dry weight)** | 74±8 | 31±2 | 24±4 | 29±5 | 16±1 | 19±1 | 86±1 | 98±2 | 91±6 | 83±6 | 97±4 | 79±14 | 29±19 | 23±7 |
| **Essential AA (% of total AA content)** | | | | | | | | | | | | | | |
| Histidine | 1.6±0.2 | 0.5±0.1 | 0.4±0.1 | 0.4±0.1 | 0.6±0.2 | 0.4±0.1 | 0.6±0.0 | 0.7±0.0 | 0.7±0.0 | 0.6±0.0 | 0.8±0.1 | 0.8±0.1 | 0.7±0.2 | 0.7±0.2 |
| Isoleucine | 4.6±0.2 | 1.4±0.1 | 1.6±0.1 | 1.5±0.1 | 1.2±0.1 | 1.5±0.1 | 1.8±0.1 | 1.6±0.1 | 1.6±0.1 | 1.3±0.1 | 1.7±0.1 | 1.5±0.1 | 2.3±0.1 | 2.2±0.1 |
| Leucine | 11.6±0.3 | 4.2±0.2 | 4.3±0.2 | 4.5±0.1 | 4.2±0.3 | 4.1±0.1 | 5.1±0.2 | 4.3±0.1 | 4.2±0.1 | 4.0±0.1 | 4.7±0.2 | 4.8±0.1 | 6.7±0.3 | 7.1±0.3 |
| Lysine | 9.3±0.4 | 2.9±0.1 | 3.1±0.1 | 3.1±0.1 | 3.4±0.1 | 3.2±0.2 | 2.8±0.1 | 2.9±0.0 | 2.8±0.1 | 3.1±0.1 | 3.5±0.1 | 3.3±0.1 | 4.6±0.2 | 4.8±0.1 |
| Methionine | 0.7±0.1 | 0.2±0.0 | 0.1±0.0 | 0.2±0.0 | 0.1±0.0 | 0.2±0.0 | 0.3±0.1 | 0.4±0.1 | 0.3±0.1 | 0.1±0.0 | 0.4±0.1 | 0.3±0.1 | 0.1±0.1 | 0.2±0.1 |
| Phenylalanine | 4.3±0.1 | 2.0±0.1 | 2.1±0.1 | 2.1±0.1 | 2.1±0.2 | 2.0±0.0 | 2.3±0.1 | 2.0±0.0 | 1.9±0.1 | 2.0±0.0 | 2.1±0.1 | 2.2±0.1 | 2.7±0.1 | 2.9±0.1 |
| Threonine | 4.6±0.2 | 1.7±0.2 | 1.8±0.2 | 1.7±0.1 | 1.6±0.1 | 1.7±0.1 | 2.1±0.1 | 1.6±0.1 | 1.5±0.1 | 1.5±0.1 | 1.8±0.2 | 1.8±0.1 | 2.3±0.1 | 2.6±0.1 |
| Valine | 5.7±0.2 | 2.7±0.2 | 3.0±0.1 | 2.7±0.1 | 3.1±0.3 | 2.9±0.1 | 2.9±0.1 | 2.9±0.1 | 2.9±0.1 | 3.1±0.1 | 3.1±0.2 | 3.4±0.1 | 4.6±0.2 | 4.8±0.2 |
| **Ʃ EAA** | **42.4** | **15.6** | **16.4** | **16.2** | **16.3** | **16.0** | **17.9** | **16.4** | **16.0** | **15.7** | **18.0** | **18.0** | **24.2** | **25.4** |
| **Non-Essential AA (% of total AA content)** | | | | | | | | | | | | | | |
| Alanine | 11.4±0.3 | 13.4±0.3 | 13.4±0.4 | 12.8±0.3 | 14.3±0.3 | 13.1±0.5 | 12.4±0.2 | 12.6±0.2 | 12.8±0.2 | 13.7±0.3 | 12.8±0.2 | 13.6±0.3 | 13.6±0.4 | 14.0±0.5 |
| Arginine | 1.8±0.0 | 1.7±0.1 | 1.9±0.0 | 1.8±0.1 | 1.6±0.1 | 1.7±0.1 | 1.9±0.0 | 1.9±0.1 | 1.8±0.0 | 1.8±0.1 | 1.8±0.0 | 1.8±0.1 | 1.7±0.1 | 1.6±0.0 |
| Aspartic acid | 7.6±0.3 | 4.0±0.3 | 3.7±0.3 | 4.0±0.2 | 3.9±0.3 | 4.0±0.2 | 4.1±0.1 | 4.0±0.2 | 3.8±0.1 | 3.9±0.1 | 4.5±0.1 | 4.3±0.1 | 5.4±0.1 | 5.5±0.2 |
| Cysteine | 0.6±0.0 | 0.2±0.0 | 0.2±0.1 | 0.2±0.1 | 0.1±0.0 | 0.1±0.0 | 0.2±0.0 | 0.2±0.0 | 0.1±0.0 | 0.1±0.0 | 0.2±0.0 | 0.2±0.0 | 0.4±0.1 | 0.5±0.0 |
| Glutamic acid | 13.1±0.3 | 7.2±0.2 | 7.7±0.3 | 7.4±0.2 | 6.7±0.2 | 7.3±0.2 | 8.0±0.3 | 6.9±0.2 | 6.6±0.1 | 6.4±0.2 | 7.1±0.1 | 6.6±0.2 | 7.1±0.4 | 7.5±0.2 |
| Glycine | 9.0±0.5 | 40.9±1.5 | 39.9±1.3 | 40.6±1.0 | 39.6±0.8 | 40.8±0.9 | 38.2±1.1 | 41.3±1.0 | 42.0±0.8 | 41.7±0.9 | 39.0±0.9 | 38.7±0.4 | 31.3±1.0 | 30.0±1.0 |
| Proline | 6.0±0.1 | 14.1±0.2 | 13.7±0.4 | 14.0±0.2 | 13.9±0.5 | 13.8±0.5 | 14.2±0.4 | 14.0±0.1 | 14.0±0.3 | 14.2±0.4 | 13.3±0.2 | 13.7±0.3 | 11.7±0.3 | 11.1±0.2 |
| Serine | 4.9±0.5 | 2.3±0.2 | 2.3±0.2 | 2.3±0.2 | 3.0±0.5 | 2.5±0.2 | 2.3±0.1 | 2.1±0.1 | 2.2±0.2 | 2.1±0.1 | 2.4±0.1 | 2.3±0.1 | 3.2±0.2 | 2.9±0.1 |
| Tyrosine | 3.3±0.1 | 0.7±0.1 | 0.8±0.1 | 0.8±0.1 | 0.6±0.0 | 0.7±0.1 | 0.8±0.0 | 0.7±0.0 | 0.7±0.0 | 0.5±0.1 | 0.9±0.1 | 0.8±0.0 | 1.4±0.1 | 1.6±0.1 |
| **Ʃ NEAA** | **57.6** | **84.4** | **83.6** | **83.8** | **83.7** | **84.0** | **82.1** | **83.6** | **84.0** | **84.3** | **82.0** | **82.0** | **75.8** | **74.6** |
